# Supplementary figures and images for: Integrated approaches to identifying cryptic bat species in areas of high endemism: The case of Rhinolophus andamanensis in the Andaman Islands
Source: PLoS One. 2019 Oct 10;14(10):e0213562. doi: 10.1371/journal.pone.0213562 (PMC6786537; doi:10.1371/journal.pone.0213562)

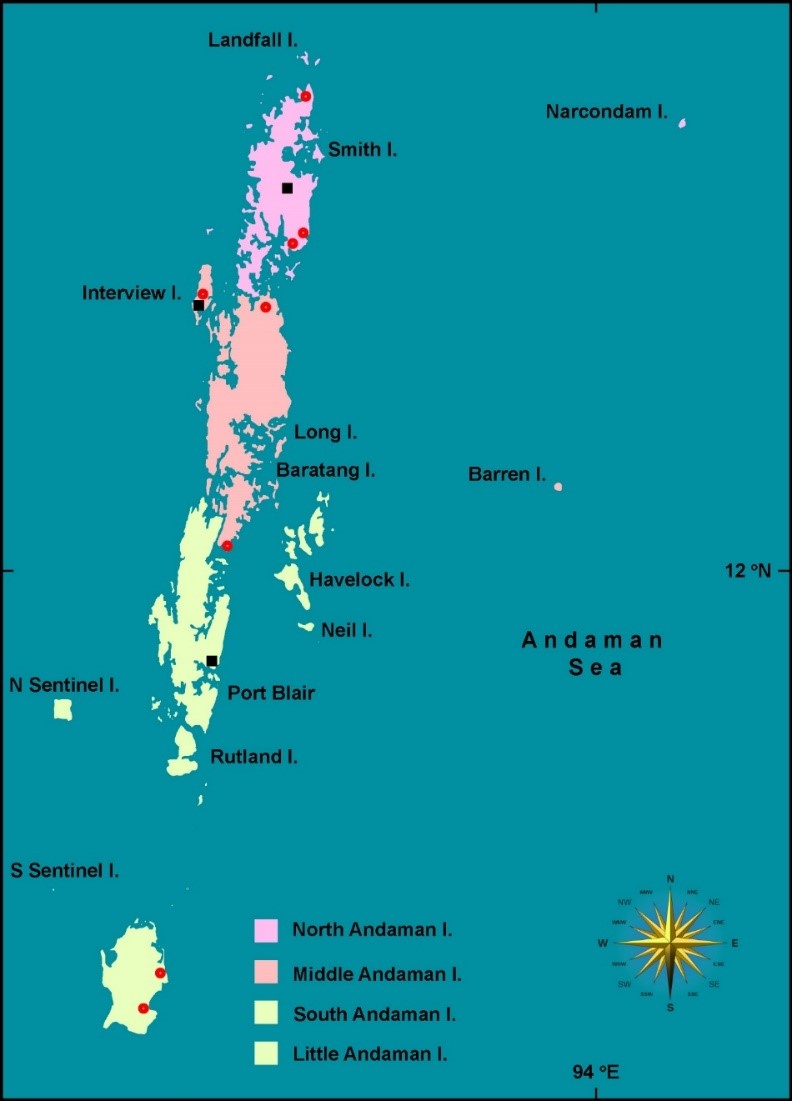

Supplement: S1 Fig — (JPG) [file pone.0213562.s001.jpg]
