# Supplementary material for: Integrated approaches to identifying cryptic bat species in areas of high endemism: The case of Rhinolophus andamanensis in the Andaman Islands
Source: PLoS One. 2019 Oct 10;14(10):e0213562. doi: 10.1371/journal.pone.0213562 (PMC6786537; doi:10.1371/journal.pone.0213562)
Supplement: S1 Table — (PDF) [file pone.0213562.s002.pdf]

S1 Table. Loading values of the first two principal components (PC1 and PC2) of the PCA analysis of external and craniodental morphometrics of *Rhinolophus andamanensis*.

**Character**	**PC1**	**PC2**
FA	2.623196	-1.0104
TL	2.104873	1.60944
EL	0.40595	0.12234
TIB	2.664489	-0.24298
HF	1.086547	-0.49199
3MT	1.402113	0.823664
4MT	1.85908	0.652603
5MT	2.429658	0.38236
1P3MT	0.946124	-0.14825
2P3MT	1.960352	-0.19593
1P4MT	0.69029	-0.13801
2P4MT	1.037403	-0.28191
GTL	1.666544	-0.47624
CCL	1.181944	-0.3175
ZB	0.5755	-0.05766
BB	0.586113	-0.21195
CM^3	0.503689	-0.15482
CM_3	0.714759	-0.3578
M	0.781093	-0.30553
M^3–M^3	0.328388	-0.09398
C^1–C^1	0.683007	-0.08832
