# Supplementary material for: Integrated approaches to identifying cryptic bat species in areas of high endemism: The case of Rhinolophus andamanensis in the Andaman Islands
Source: PLoS One. 2019 Oct 10;14(10):e0213562. doi: 10.1371/journal.pone.0213562 (PMC6786537; doi:10.1371/journal.pone.0213562)
Supplement: S2 Table — (DOCX) [file pone.0213562.s003.docx]

**S2 Table. Cytochrome C oxidase subunit 1 (COI) sequences of *Rhinolophus andamanensis* and *Rhinolophus affinis*, their collection localities, and GenBank accession numbers used for conducting the phylogenetic analysis; *Rhinolophus lepidus* was used as outgroup taxon.**

| **S. No.** | **Species** | **Collection locality** | **Accession number** |
| --- | --- | --- | --- |
| 1. | *R. andamanensis* | Interview Island, Andaman Islands | MG821184 |
| 2. | *R. andamanensis* | Baratang Island, Andaman Island | MG821169 |
| 3. | *R. andamanensis* | Pathilevel, Middle Andaman, Andaman Islands | MG821172 |
| 4. | *R. andamanensis* | V.K. Pur Little Andaman, Andaman Islands | MG821181 |
| 5. | *R. andamanensis* | Chipo, North Andaman | MG821178 |
| 6. | *R. affinis* | Hala Bala, Thailand | KP192675 |
| 7. | *R. affinis* | Hala Bala, Thailand | KP192676 |
| 8. | *R. affinis* | Thailand | KP192677 |
| 9. | *R. affinis* | Thailand | KP192678 |
| 10. | *R. affinis* | Adang, Thailand | KP192691 |
| 11. | *R. affinis* | Adang, Thailand | KP192692 |
| 12. | *R. affinis* | Johor, Kuala Jasin, Endau Rompin National Park, Malaysia | HM541413 |
| 13. | *R. affinis* | Negeri Sembilan, Pasoh Forest Reserve, Malaysia | HM541331 |
| 14. | *R. affinis* | Myanmar (Southern) | KP192682 |
| 15. | *R. affinis* | Myanmar (Northern) | HM541326 |
| 16. | *R. affinis* | Guangxi, Jing Xin County Provincial Nature Reserve, China | HM541393 |
| 17. | *R. affinis* | Guangxi, Shiwandashan National Reserve, China | HM541354 |
| 18. | *R. affinis* | Hunan, Dongnam, Shuhuangshan Reserve, China | JF444035 |
| 19. | *R. affinis* | Viet Nam | KP192685 |
| 20 | *R. affinis* | Viet Nam | KP192686 |
| 21. | *R. affinis* | Xiangkhoang, Nam Et NBCA, north of Ban Houay Kho, Laos | HM541336 |
| 22. | *R. affinis* | Louang Namtha, Outskirts of Ban Phoulan, Laos | HM541339 |
| 23. | *R. lepidus* | Dong Nai, Cat Tien National Park, Cat Tien National Park Headquarters, Viet Nam | HM541577 |
| 24. | *R. lepidus* | Dong Nai, Than Phu, Cat Tien National Park, Nam Cat Tien, Viet Nam | HM541581 |
